# GFR estimation is complicated by a high incidence of non-steady-state serum creatinine concentrations at the emergency department

**DOI:** 10.1371/journal.pone.0261977

**Published:** 2021-12-29

**Authors:** M. S. A. Niemantsverdriet, T. T. Pieters, I. E. Hoefer, M. C. Verhaar, J. A. Joles, W. W. van Solinge, W. M. Tiel Groenestege, S. Haitjema, M. B. Rookmaaker

**Affiliations:** 1 Central Diagnostic Laboratory, University Medical Center Utrecht, Utrecht University, Utrecht, The Netherlands; 2 SkylineDx, Rotterdam, The Netherlands; 3 Department of Nephrology and Hypertension, University Medical Center Utrecht, Utrecht University, Utrecht, The Netherlands; University of Colorado Denver School of Medicine, UNITED STATES

## Abstract

**Background:**

Acquiring a reliable estimate of glomerular filtration rate (eGFR) at the emergency department (ED) is important for clinical management and for dosing renally excreted drugs. However, renal function formulas such as CKD-EPI can give biased results when serum creatinine (SCr) is not in steady-state because the assumption that urinary creatinine excretion is constant is then invalid. We assessed the extent of this by analysing variability in SCr in patients who visited the ED of a tertiary care centre.

**Methods:**

Data from ED visits at the University Medical Centre Utrecht, the Netherlands between 2012 and 2019 were extracted from the Utrecht Patient Oriented Database. Three measurement time points were defined for each visit: last SCr measurement before visit as baseline (SCr-BL), first measurement during visit (SCr-ED) and a subsequent measurement between 6 and 24 hours during admission (SCr-H1). Non-steady-state SCr was defined as exceeding the Reference Change Value (RCV), with 15% decrease or 18% increase between successive SCr measurements. Exceeding the RCV was deemed as a significant change.

**Results:**

Of visits where SCr-BL and SCr-ED were measured (N = 47,540), 28.0% showed significant change in SCr. Of 17,928 visits admitted to the hospital with a SCr-H1 after SCr-ED, 27,7% showed significant change. More than half (55%) of the patients with SCr values available at all three timepoints (11,054) showed at least one significant change in SCr over time.

**Conclusion:**

One third of ED visits preceded and/or followed by creatinine measurement show non-stable serum creatinine concentration. At the ED automatically calculated eGFR should therefore be interpreted with great caution when assessing kidney function.

## Introduction

Assessment of kidney function plays a crucial role in the evaluation and treatment of patients. A change in kidney function can point to renal disease, which is associated with an increase in morbidity and mortality [1, 2]. Timely therapeutic intervention may attenuate or prevent renal damage [3, 4]. Furthermore, kidney function is essential for drug dosing, ensuring optimal efficacy while reducing potential toxicity [5, 6].

Renal function is most often quantified as the estimated glomerular filtration rate (eGFR), calculated by the CKD-EPI formula using serum creatinine (SCr), age, gender, and race [7]. The CKD-EPI formula was developed in patients with chronic kidney disease and a stable kidney function. However, in patients with changes in renal function due to, for example acute kidney injury (AKI), it takes time before SCr has reached its new steady-state because the assumption that urinary creatinine excretion is constant is then invalid [8, 9]. In these situations, the CKD-EPI is inaccurate and lags behind the true eGFR for up to 3 days [10]. As AKI is frequently seen at the emergency department (ED), CKD-EPI, as well as older formulas used to calculate eGFR such as MDRD, might often not adequately estimate the actual GFR at the ED.

Dosing of renally cleared drugs is often based on KDIGO CKD-categories using a measure of eGFR such as the CKD-EPI. When SCr is not in steady-state, drug dosing based on the CKD-EPI may result in potential toxicity or underdosing. Indeed, up to 24% of admitted patients with AKI experience some form of adverse event caused by inadequate drug dosing [6].

In this study we aimed to assess the incidence of non-steady-state SCr concentrations at the ED and therefore situations where the CKD-EPI is potentially unreliable. We hypothesized that a substantial number of patients who visit the emergency department an in whom a serum creatinine concentration is assessed have a serum creatinine concentration that is not in steady-state.

## Materials and methods

### Study design and population (data extraction)

We performed a single centre retrospective analysis, using data from the University Medical Centre (UMC) Utrecht, Utrecht, the Netherlands. We evaluated all ED visits between 2012 and 2019 from patients aged over 18 years. Data were extracted from the Utrecht Patient Oriented Database (UPOD) [11]. In brief, UPOD is an infrastructure of relational databases comprising data on patient characteristics, hospital discharge diagnoses, medical procedures, medication orders and laboratory tests for all patients treated at the UMC Utrecht since 2004.

For each ED visit we extracted patient age, sex and hospitalization information. Additionally, all SCr measurements were extracted 365 days prior and up to 24 hours after each ED visit. SCr was measured by enzymatic colorimetric assay (*Beckman Coulter*, *Brea*, *CA*, *USA*). For each SCr an eGFR was computed using the CKD-EPI formula [7]. Chronic kidney disease (CKD) was defined by the KDIGO 2012 criteria based on the estimated GFR [12].

### Definition of serum creatinine not in steady-state

We defined the following three SCr measurements: baseline SCr measurement as the most recent SCr measurement within a year before ED presentation (SCr-BL), SCr-ED as the measurement at ED presentation, and a subsequent SCr measurement during hospitalization (SCr-H1). Since very short time-intervals (e.g. 30 minutes) may obscure significant fluctuations in SCr, we defined SCr-H1 as the first measurement closest to 12 hours and at least 6 hours with a maximum of 24 hours after the SCr-ED measurement.

Significant fluctuation in SCr was defined as exceeding the Reference Change Value (RCV). RCV represents the smallest difference between sequential laboratory results representing a true change in the patient and can be calculated using the analytical coefficient of variation (CV_a_) and within-subject biological coefficient of variation (CV_i_). Since SCr does not follow a normal distribution due to the underlying first-order elimination, we calculated the RCV using the log-method: $\sigma =\sqrt{ln(CV_{i}^{2}+CV_{a}^{2}+1)},\;RCV=e^{+/-1,96*\sqrt{2}*\sigma }$. With this method, we calculated the RCV (CV_i_ = 5.95% and CVa = 1%) resulting in a significant increase of 18% and a significant decrease of 15% [13, 14].

Since drug dosing is often based on CKD-EPI categories (e.g. <15 ml/min/1.73m^2^, 15–30 ml/min/1.73m^2^, 30–45 ml/min/1.73m^2^ etc.), we also assessed the change in CKD-EPI categories between baseline measurement and ED measurement as well as baseline and the subsequent measurement during admission [15].

### Ethics

This study was performed according to the declaration of Helsinki and the ethical guidelines of our institution. The institutional review board of the UMC Utrecht waived the need for informed consent. Pseudonymized data were used for this study. Data collection and handling was conducted in accordance with European privacy legislation (GDPR).

### Statistics

Risk of non-steady-state SCr was quantified with logistic regression. 95% confidence intervals for the absolute change in creatinine over time were calculated with smoothed quantile regression. All statistics and pre-processing were performed using the R environment (3.6.1). P-values below 0.05 were considered significant.

## Results

### Patient characteristics

Between 2012 and 2019 there were 120,652 visits from 69,579 unique patients who visited the ED (S1 Fig and Table 1). Of all visits, there were slightly more males (54%) than females (46%) and the average age was 53.8 years. Three not mutually exclusive groups were defined for analysis: visits with a SCr-BL and SCr-ED measurement (N = 47,540), visits with a SCr-ED and SCr-H1 measurement (N = 17,928), and visits with measurement at all timepoints (N = 11,054) (S1 Fig, S1 Table).

**Table 1:** Characteristics of all emergency department (ED) visits.

Unique ED visits	120,652
Age, years mean (SD)	53.8 (19.2)
Unique patients	69,579
Male sex, count (%)	65,107 (54.0%)
Admitted, count (%)	58,154 (48.2%)
CKD category at ED, % (n)	
G1	36,075 (29.9%)
G2	26,757 (22.2%)
G3a	7,875 (6.5%)
G3b	5,293 (4.4%)
G4	3,385 (2.8%)
G5	2,026 (1.7%)
missing	39,241 (32.5%)
ED discipline, % (n)	
Cardiology	16,706 (13.8%)
Gastroenterology	4,318 (3.6%)
Internal medicine	24,766 (20.5%)
Lung	7,643 (6.3%)
Nephrology	2,747 (2.3%)
Neurology	16,882 (14.0%)
Other	6,667 (5.5%)
Surgical	36,766 (30.5%)
Urology	4,157 (3.4%)
SCr-BL, μmol/L, mean (SD)	100.3 (104.4)
Missing, count (%)	58,944 (48.9%)
SCr-ED, μmol/L, mean (SD)	98.9 (100.9)
Missing, count (%)	39,241 (32.5%)
SCr-H1, μmol/L, mean (SD)	126.0 (137.6)
Missing, count (%)	101,452 (84.1%)

CKD severity was not computed for 39,241 ED visits with no serum creatinine (SCr) available at visit.

Percentages are based on the total number of visits. ED discipline was defined as the first discipline the patient visited during visit.

### Incidence of serum creatinine concentration not in steady-state at the emergency department as compared to baseline

**Fig 1:**
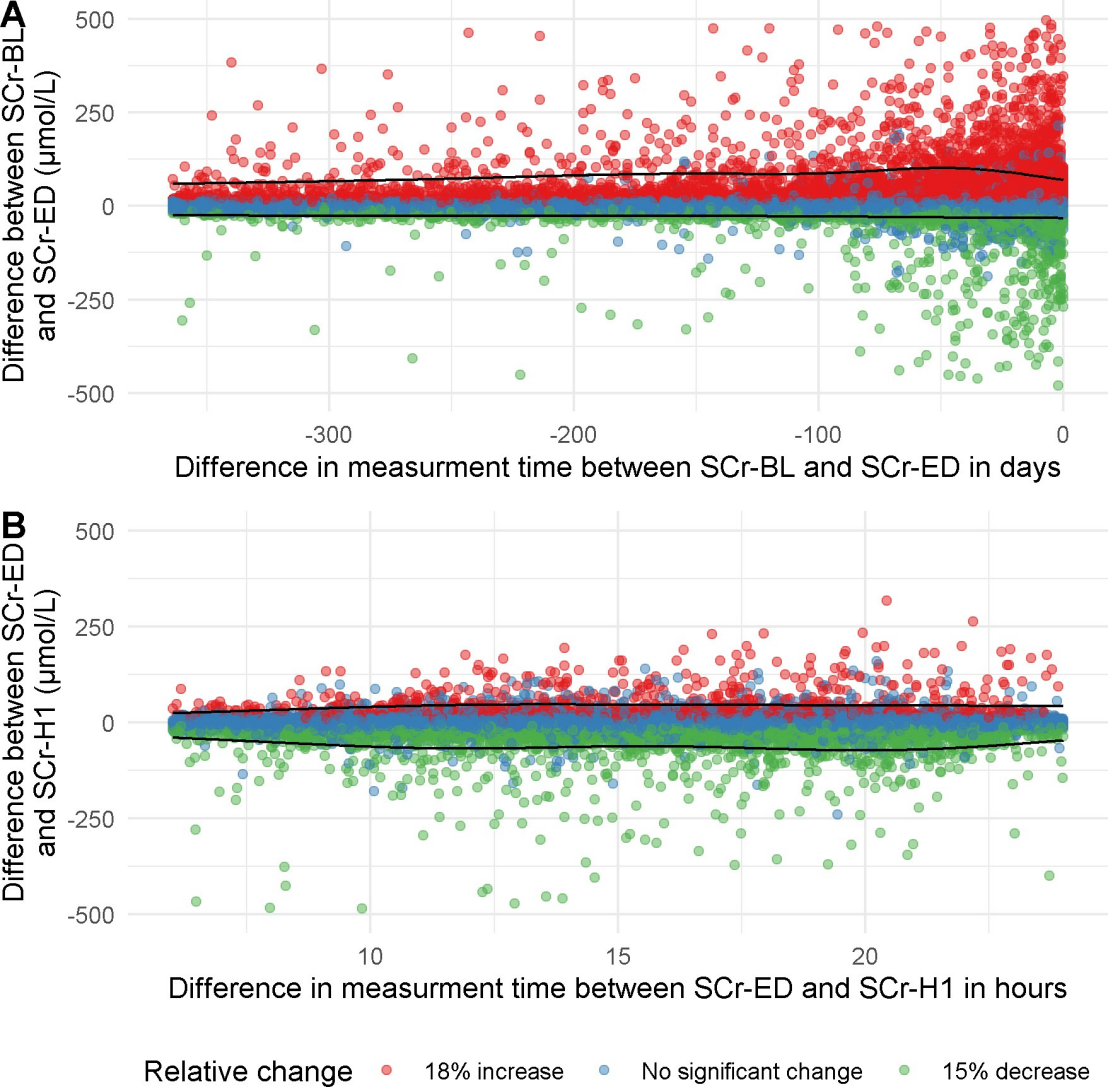
Absolute and relative difference in creatinine between the baseline measurement (SCr-BL) and the creatinine measurement at emergency department presentation (SCr-ED) (A) and between SCr-ED and the first subsequent creatinine measurement after hospitalisation (SCr-H1) (B) compared to the difference in days between measurements. Colours indicate a 18% increase (red), 15% decrease (green) or no significant change (blue). The black lines represent the smoothed 95% confidence interval calculated with quantile regression.

When SCr-ED was compared to SCr-BL, 8,794 visits (18.5%) showed a significant increase and 5,378 (11.3%) showed a significant decrease SCr (Fig 1A). The median time between baseline and ED was 26.8 days with an interquartile range of 8.4 to 83.0 hours. The number of patients with a significant change in serum creatinine decreased when time between SCr-BL and SCr-ED was longer (S2 Fig). 29.8% of these visits changed at least one CKD category (S2 Table).

### Incidence of serum creatinine concentration not in steady-state at the emergency department as compared to follow-up measurements

Of the patients with both a SCr-ED and a SCr-H1, a significant increase was seen in 1,304 (7.3%) and a significant decrease was seen in 3,839 subjects (21.4%) (Fig 1B). The median time between ED presentation and the subsequent measurement was 15.7 hours with an interquartile range of 12.0 to 19.1 hours. 27.7% of these visits changed at least one CKD category (S3 Table).

### Consistency of serum creatinine change over time

Next, we compared the consistency of SCr in visits with all three measurements available (SCr-BL, SCr-ED and SCr-H1, N = 11,054). After a significant rise in SCr between BL and ED (N = 3,993), 4.4% continued to rise significantly, 58.7% stabilised, and 36.9% had a significant decrease in SCr (Table 2). After a significant decrease in SCr between BL and ED (N = 1,016), 5.7% continued to decrease, 76.4% stabilised, and 17.9% showed a significant increase in SCr. Despite having a stable SCr between BL and ED (N = 6,045), a significant rise was seen in 6.3% and a significant decrease was seen in 11.4% of these admissions. Taken together, more than half (55%) of the patients of whom SCr values were available for all three timepoints, showed at least one significant change in Scr and were therefore not in steady state.

**Table 2:** Relative changes of serum creatinine (SCr) measurement between baseline (BL), emergency department (ED) and the SCr measured during hospitalization (H1).

BL-ED ED-H1	15% decrease	No significant change	18% increase	Cumulative
15% decrease	58 (0.5%)	690 (6.2%)	1,473 (13.3%)	2,221 (20.0%)
No significant change	776 (7.0%)	4,972 (45.0%)	2,345 (21.2%)	8,093 (73.2%)
18% increase	182 (1.7%)	383 (3.5%)	175 (1.6%)	740 (6.8%)
Cumulative	1,016 (9.2%)	6,045 (54.7%)	3,993 (36.1%)	11,054 (100%)

The table shows relative changes for 11,054 ED visits with SCr-BL, SCr-ED and SCr-H1 measurements. BL-ED represents the difference between SCr-BL and SCr-ED, whereas ED-H1 represents the difference between SCr-ED and SCr-H1.

### Incidence of serum creatinine not in steady-state between medical specialties and CKD stages

We compared the incidence for non-steady-state SCr between different medical specialties and CKD-stages. Visits with CKD stage 3a or worse were at a higher risk of non-steady-state SCr between SCr-BL and SCr-ED (p<0.001; S4 Table). Similarly, when comparing SCr-ED to SCr-H1 visits with CKD stage 2 or worse (except for CKD stage 5) were at a higher risk of non-steady-state SCr (p<0.001; S4 Table).

**Fig 2:**
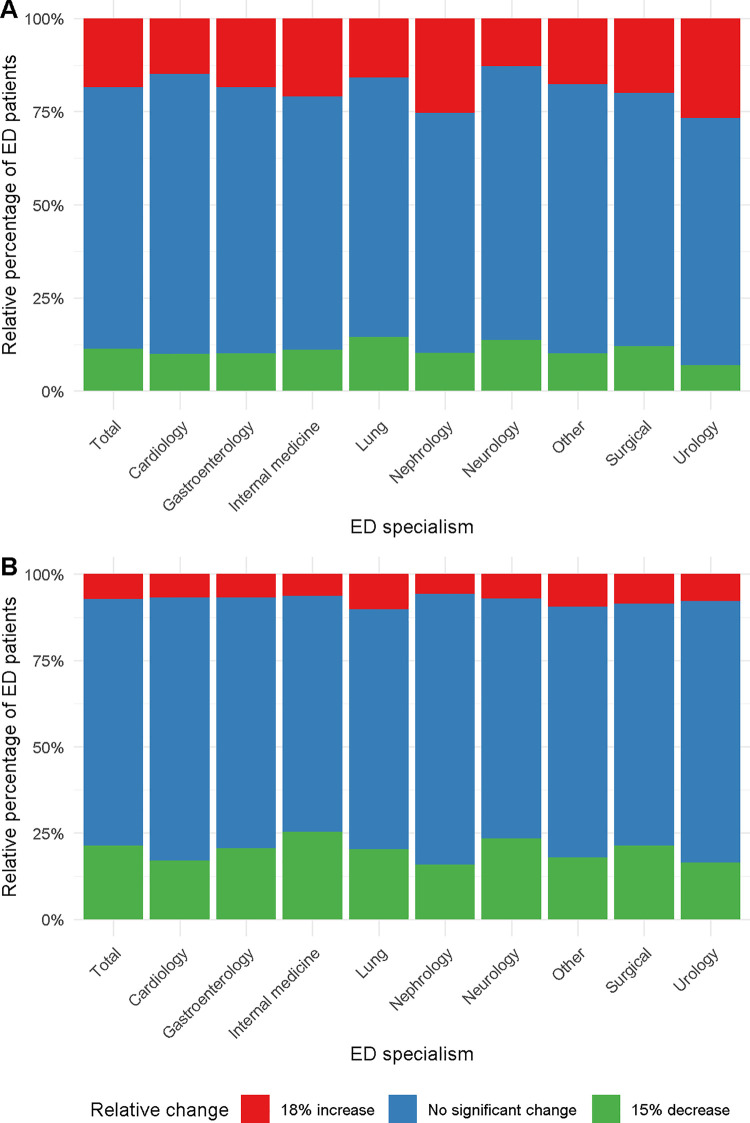
Relative percentages of relative change per emergency department (ED) specialism for all ED visits with a baseline SCr measurement and a SCr measurement at ED visit (N = 47,540) (A) and all visits with a SCr measurement at ED visit and a subsequent SCr measurement after hospital admission (N = 17,928) (B). Colors indicate a 18% increase (red), 15% decrease (green) or no significant change (blue).

Next, we compared the incidence of a non-steady-state creatinine between different medical specialties at the ED, as one might hypothesize that this phenomenon preferentially occurs in certain specialties. Although the percentage of non-steady state creatinine concentrations differed between the different specialties (Fig 2; S6 and S7 Tables), the incidence was substantial (22%-36%) in all specialties.

## Discussion

Reliable estimation of the GFR at the ED is the cornerstone of assessing renal function and thereby essential for correct dosing of drugs that are renally excreted. However, commonly used renal function formulas (CKD-EPI, MDRD) require SCr in steady-state to provide a reliable estimate of the GFR. We found that a third of all the SCr measured at our ED were not in steady-state and that the CKD-EPI may therefore not reflect the underlying renal function. Faulty GFR estimates and CKD staging will affect clinical decision making and drug dosing regimens. Interestingly there appeared to be an inverse correlation between the incidence of non-steady state creatinine concentration and elapsed time since last creatinine concentration measurement before the ED measurement. We found a similar high incidence of non-steady-state SCr across all different specialities.

The incidence of non-steady-state SCr and the reliability of the CKD-EPI at the ED has not been widely studied. Previous reports have estimated the incidence of AKI in the general ED-population between 3% and 25%, depending on the definition and the population [16, 17]. One study that investigated non-steady-state SCr in patients that were admitted to the hospital after ED visit found that nearly half of the visits had non-steady-state SCr during the entire length of hospital stay [18]. To our knowledge we are the first to report the high incidence of a non-steady-state SCr at the ED, which raises serious concerns about the applicability of eGFR formulas like CKD-EPI at the ED.

The high incidence of SCr not in steady-state in the ED not only has consequences for the interpretation of the CKD-EPI as a measure of kidney function per se, but also for dosing of drugs with a significant renal clearance, since this is often guided by CKD categories indexed by the eGFR. The CKD-EPI based eGFR calculated during ED visit and subsequent admission results in frequently changing CKD categories over time (27,7–29,8%). Moreover, an increase or decrease in CKD-EPI categories from baseline to ED was not associated with a subsequent increase or decrease in CDK-EPI categories from ED to H1. Although the changing CKD categories do reflect changes in underlying renal function, they do not adequately reflect the actual GFR and should therefore be used with caution for dosing of drugs with significant renal clearance.

Most formulas that estimate glomerular filtration rate assume a stabilized serum creatinine concentration, and therefore only require the input of one serum creatinine concentration value [19, 20]. Performing a consecutive SCr measurement will provide insight in serum creatinine fluctuation. Moreover, this consecutive SCr can be used for alternative approaches that have been published that can be used to estimate renal function when SCr is not in steady-state [21]. These “kinetic” formulas have been applied in patients admitted to the intensive care and in patients after kidney transplantation and calculate the eGFR by combining two SCr measurements with an estimate of the production and volume of distribution of creatinine [8, 9, 22]. Although physiologically interesting, these formulas have not been rigorously validated in patients at the ED. In the future, use of thresholds calculated with the RCV may help identify patients with a SCr not in steady state, where “kinetic” eGFR formulas may give a better estimation of true underlying GFR.

There are other instances where serum creatinine based renal function formulas may be inaccurate. These are, amongst others, situations that influence the production or excretion of creatinine independent of GFR, such as aberrant diet, pregnancy, skeletal muscle disease and drugs that influence tubular secretion of creatinine. This further stresses the limitation single serum creatinine concentration based renal function formulas to estimate renal function at the ED. It is of note that cystatin C based renal function formulas have been proposed to be less dependent on muscle mass and diet. However, current cystatin c based renal function formulas also require steady state serum concentrations of cystatin C to estimate renal function from a single serum cystatin C concentration.

Strengths of this study are that we used a large dataset with a well-documented and unselected population. This allowed us to study the true incidence of non-steady-state SCr at the ED of our tertiary care hospital over different medical specialities and CKD stages. The detailed time annotation allowed us to not only study non-steady-state SCr between baseline and the ED measurement but also the subsequent measurement within 24h after admission. Furthermore, the use of a relational database such as UPOD ensures maximum completeness and integrity of the data, since it continuously stores laboratory and clinical data for every individual ED visit. This allowed us to perform our analyses on routine care data that is a valid representation how the CKD-EPI formula is used in clinical practice.

This study has some drawbacks. This retrospective study only included ED patients in whom creatinine concentrations were determined, which introduced selection. Although this might lead to overestimation of non-steady state creatinine concentrations at the ED population as a whole, it does reflect the incidence of non-steady state creatinine concentration in the ED subpopulation where renal function estimation is deemed appropriate by the treating physician. Another drawback of this retrospective cohort study is the lack of invasive GFR measurements to assess actual GFR. Although currently no validated formula is available to estimate underlying renal function in patients with a non-steady state serum creatinine concentration at the ED, the simple notion that an increasing serum creatinine concentration causes the CKD-EPI to underestimate underlying renal function (and vice versa) is important and may be an incentive to better estimate renal function with timed urine collections. Finally, the current study was neither designed to show any adverse clinical consequences of the faulty GFR estimates nor to quantify potential benefits of improved GFR estimates. However, the abovementioned high percentage of adverse drug reaction due to inadequate dosing in patients suffering from AKI, suggests room for improvement.

In conclusion, a third of the patients who visit the ED have non-steady-state SCr. Physicians should be aware of this when using the automatically provided CKD-EPI at the ED and should interpret the reported eGFR with great caution. Future studies should elucidate whether a more tailored GFR estimate (e.g. by using dynamic formulas or timed urine collections) improve drug dosing and/or clinical outcome.

## Supporting information

S1 Table Characteristics of the three emergency department (ED) sub-cohorts depended on availability of serum creatinine (SCr) measurement, not mutually exclusive. Percentages are based on the total number of visits per sub-cohort. ED discipline was defined as the first discipline the patient visited during visit. ICU admission was defined as having at least one admission to the ICU during hospital stay.
(DOCX)

S2 Table CKD staging changes between the baseline eGFR (CKD-BL) and the eGFR at emergency department (CKD-ED). (DOCX)

S3 Table CKD staging changes between the emergency department eGFR (CDK-ED) and the subsequent eGFR during visit (CKD-H1). (DOCX)

S4 Table Odds ratio for each CKD-EPI stage based on SCr-BL compared with the G1 CKD-EPI stage in respect to a non-steady-state serum creatinine (SCr) between SCr-BL and SCr-ED. (DOCX)

S5 Table Odds ratio for each CKD-EPI stage based on SCr-ED compared with the G1 CKD-EPI stage in respect to a non-steady-state serum creatinine (SCr) between SCr-ED and SCr-H1. (DOCX)

S6 Table Odds ratio for each emergency department (ED) specialism compared with the nephrology ED specialism in respect to a non-steady-state serum creatinine (SCr) between SCr-BL and SCr-ED. *: p-value < 0.001.
(DOCX)

S7 Table Odds ratio for each emergency department (ED) specialism compared with the nephrology ED specialism in respect to a non-steady-state serum creatinine (SCr) between SCr-ED and SCr-H1. *: p-value < 0.001.
(DOCX)

S1 Fig Overview of the number of emergency department (ED) visits with serum creatinine (SCr) measurements in their clinical history at specific time points. Percentages computed from the total number of ED visits.
(TIFF)

S2 Fig Number of patients with a significant increase or significant decrease between the baseline measurement (SCr-BL) and the creatinine measurement at emergency department presentation (SCr-ED). Patients are grouped based on the time to last creatinine measurement before emergency department (ED) visit.
(TIFF)
