# Supplementary material for: GFR estimation is complicated by a high incidence of non-steady-state serum creatinine concentrations at the emergency department
Source: PLoS One. 2021 Dec 29;16(12):e0261977. doi: 10.1371/journal.pone.0261977 (PMC8716053; doi:10.1371/journal.pone.0261977)
Supplement: S1 Table — Percentages are based on the total number of visits per sub-cohort. ED discipline was defined as the first discipline the patient visited during visit. ICU admission was defined as having at least one admission to the ICU during hospital stay. (DOCX) [file pone.0261977.s001.docx]

**S1 Table: Characteristics of the three emergency department (ED) sub-cohorts depended on availability of serum creatinine (SCr) measurement, not mutually exclusive.** Percentages are based on the total number of visits per sub-cohort. ED discipline was defined as the first discipline the patient visited during visit. ICU admission was defined as having at least one admission to the ICU during hospital stay.

|  | BL-ED cohort | ED-H1 cohort | BL-ED-H1 cohort |
| --- | --- | --- | --- |
| Unique ED visits  Age, years mean (SD) | 47,540 59.0 (16.8) | 17,928 61.3 (16.9) | 11,054  61.7 (16.0) |
| Unique patients | 20,608 | 13,218 | 7,089 |
| Male sex, count (%) | 25,438 (53.5%) | 10,171 (56.7%) | 6,174 (55.9%) |
| Admitted, count (%) | 29,751 (62.6%) | 17,573 (98.0%) | 10,799 (97.7%) |
| Hospital length in days, mean (SD) | 6.7 (10.0) | 10.1 (13.7) | 9.0 (11.8) |
| ICU admission during hospital stay | 1,625 (5.5%) | 3,527 (20.1%) | 1,095 (10.1%) |
| CKD category at ED, % (n) G1  G2  G3a  G3b  G4  G5 | 18,322 (38.5%)  15,542 (32.7%)  5,316 (11.2%)  3,977 (8.4%)  2,752 (5.8%)  1,631 (3.4%) | 5,142 (28.7%)  5,144 (28.7%)  2,528 (14.1%)  2,218 (12.4%)  1,843 (10.3%)  1,053 (5.9%) | 2,568 (23.2%)  2,817 (25.5%)  1,727 (15.6%)  1,657 (15.0%)  1,484 (13.4%)  801 (7.2%) |
| ED discipline, % (n)  Cardiology  Gastroenterology  Internal medicine  Lung  Nephrology  Neurology  Other  Surgical  Urology | 9,035 (19.0%)  2,892 (6.1%)  15,281 (31.1%)  5,366 (11.3%)  2,269 (4.8%)  5,227 (11.0%)  546 (1.1%)  4,746 (10.0%)  2,178 (4.6%) | 2,931 (16.3%)  948 (5.3%)  5,300 (29.6%)  1,290 (7.2%)  1,044 (5.8%)  2,279 (12.7%)  190 (1.1%)  3,086 (17.2%)  860 (4.8%) | 1,718 (15.5%)  751 (6.8%)  4,154 (37.6%)  1,046 (9.5%)  914 (8.3%)  532 (4.8%)  111 (1.0%)  1,104 (10.0%)  724 (6.5%) |
| Deceased during hospital stay | 2,707 (5.7%) | 1,921 (10.7%) | 1,061 (9.6%) |
| SCr-BL, µmol/L, mean (SD)  Missing, count (%) | 102.1 (106.5)  0 (0.0%) | 121.6 (122.0)  6,874 (38.3%) | 121.6 (122.0)  0 (0.0%) |
| SCr-ED, µmol/L, mean (SD)  Missing, count (%) | 108.2 (113.0)  0 (0.0%) | 132.6 (143.6) 0 (0.0%) | 146.0 (146.8)  0 (0.0%) |
| SCr-H1, µmol/L, mean (SD)  Missing, count (%) | 139.0 (142.5)  36,486 (76.7%) | 125.9 (137.0) 0 (0.0%) | 139.0 (142.5)  0 (0.0%) |
