# Supplementary material for: GFR estimation is complicated by a high incidence of non-steady-state serum creatinine concentrations at the emergency department
Source: PLoS One. 2021 Dec 29;16(12):e0261977. doi: 10.1371/journal.pone.0261977 (PMC8716053; doi:10.1371/journal.pone.0261977)
Supplement: S2 Table — (DOCX) [file pone.0261977.s002.docx]

S1 Table. CKD staging changes between the baseline eGFR (CKD-BL) and the eGFR at emergency department (CKD-ED).

| CKD-ED  CKD-BL | G1 | G2 | G3a | G3b | G4 | G5 |
| --- | --- | --- | --- | --- | --- | --- |
| G1 | 15,625 (32.9%) | 2,896 (6.1%) | 209 (0.4%) | 102 (0.2%) | 57 (0.1%) | 21 (0.0%) |
| G2 | 2,667 (5.6%) | 11,336 (23.8%) | 1,947 (4.1%) | 527 (1.1%) | 167 (0.4%) | 53 (0.1%) |
| G3a | 25 (0.1%) | 1,235 (2.6%) | 2,565 (5.4%) | 1,117 (2.3%) | 201 (0.4%) | 44 (0.1%) |
| G3b | 3 (0.0%) | 69 (0.1%) | 578 (1.2%) | 1,977 (4.2%) | 712 (1.5%) | 61 (0.1%) |
| G4 | 1 (0.0%) | 6 (0.0%) | 16 (0.0%) | 252 (0.5%) | 1,529 (3.2%) | 270 (0.6%) |
| G5 | 1 (0.0%) | 0 (0.0%) | 1 (0.0%) | 2 (0.0%) | 86 (0.2%) | 1,182 (2.5%) |
