# Supplementary material for: GFR estimation is complicated by a high incidence of non-steady-state serum creatinine concentrations at the emergency department
Source: PLoS One. 2021 Dec 29;16(12):e0261977. doi: 10.1371/journal.pone.0261977 (PMC8716053; doi:10.1371/journal.pone.0261977)
Supplement: S3 Table — (DOCX) [file pone.0261977.s003.docx]

S2 Table. CKD staging changes between the emergency department eGFR (CDK-ED) and the subsequent eGFR during visit (CKD-H1).

| CKD-H1  CKD-ED | G1 | G2 | G3a | G3b | G4 | G5 |
| --- | --- | --- | --- | --- | --- | --- |
| G1 | 4,687 (26.1%) | 429 (2.4%) | 19 (0.1%) | 6 (0.0%) | 1 (0.0%) | 0 (0.0%) |
| G2 | 1,204 (6.7%) | 3,505 (19.6%) | 372 (2.1%) | 56 (0.3%) | 7 (0.0%) | 0 (0.0%) |
| G3a | 76 (0.4%) | 905 (5.0%) | 1,267 (7.1%) | 255 (1.4%) | 25 (0.1%) | 0 (0.0%) |
| G3b | 20 (0.1%) | 152 (0.8%) | 566 (3.2%) | 1,301 (7.3%) | 178 (1.0%) | 1 (0.0%) |
| G4 | 5 (0.0%) | 20 (0.1%) | 42 (0.2%) | 421 (2.3%) | 1,309 (7.3%) | 46 (0.3%) |
| G5 | 0 (0.0%) | 2 (0.0%) | 0 (0.0%) | 12 (0.1%) | 139 (0.8%) | 900 (5.0)% |
