# Supplementary material for: GFR estimation is complicated by a high incidence of non-steady-state serum creatinine concentrations at the emergency department
Source: PLoS One. 2021 Dec 29;16(12):e0261977. doi: 10.1371/journal.pone.0261977 (PMC8716053; doi:10.1371/journal.pone.0261977)
Supplement: S4 Table — (DOCX) [file pone.0261977.s004.docx]

S3 Table. Odds ratio for each CKD-EPI stage based on SCr-BL compared with the G1 CKD-EPI stage in respect to a non-steady-state serum creatinine (SCr) between SCr-BL and SCr-ED.

| CKD-stage at baseline | Odds ratio | 95% CI lower bound | 95% CI upper bound |
| --- | --- | --- | --- |
| G1 vs G2 | 1.004 | 0.959 | 1.051 |
| G1 vs G3a | 1.229 | 1.151 | 1.313 |
| G1 vs G3b | 1.254 | 1.169 | 1.365 |
| G1 vs G4 | 1.291 | 1.173 | 1.421 |
| G1 vs G5 | 1.528 | 1.358 | 1.718 |
