# Supplementary material for: GFR estimation is complicated by a high incidence of non-steady-state serum creatinine concentrations at the emergency department
Source: PLoS One. 2021 Dec 29;16(12):e0261977. doi: 10.1371/journal.pone.0261977 (PMC8716053; doi:10.1371/journal.pone.0261977)
Supplement: S5 Table — (DOCX) [file pone.0261977.s005.docx]

S4 Table. Odds ratio for each CKD-EPI stage based on SCr-ED compared with the G1 CKD-EPI stage in respect to a non-steady-state serum creatinine (SCr) between SCr-ED and SCr-H1.

| CKD-stage at ED visit | Odds ratio | 95% CI lower bound | 95% CI upper bound |
| --- | --- | --- | --- |
| G1 vs G2 | 1.183 | 1.083 | 1.291 |
| G1 vs G3a | 1.460 | 1.316 | 1.621 |
| G1 vs G3b | 1.498 | 1.344 | 1.670 |
| G1 vs G4 | 1.319 | 1.173 | 1.483 |
| G1 vs G5 | 1.044 | 0.897 | 1.215 |
