# Supplementary material for: GFR estimation is complicated by a high incidence of non-steady-state serum creatinine concentrations at the emergency department
Source: PLoS One. 2021 Dec 29;16(12):e0261977. doi: 10.1371/journal.pone.0261977 (PMC8716053; doi:10.1371/journal.pone.0261977)
Supplement: S6 Table — *: p-value < 0.001. (DOCX) [file pone.0261977.s006.docx]

S5 Table. Odds ratio for each emergency department (ED) specialism compared with the nephrology ED specialism in respect to a non-steady-state serum creatinine (SCr) between SCr-BL and SCr-ED. *: p-value < 0.001

| ED specialism | Odds ratio | 95% CI  lower bound | 95% CI  upper bound | Percentage  non-steady-state SCr |
| --- | --- | --- | --- | --- |
| Intercept (nephrology)* | 1.000 |  |  | 35.7% |
| cardiology | 0.597 | 0.541 | 0.659 | 24.9% |
| gastroenterology* | 0.718 | 0.638 | 0.808 | 28.5% |
| internal medicine | 0.848 | 0.773 | 0.930 | 32.0% |
| lung* | 0.787 | 0.710 | 0.874 | 30.4% |
| neurology | 0.650 | 0.585 | 0.722 | 26.5% |
| other* | 0.690 | 0.560 | 0.846 | 27.7% |
| surgical | 0.845 | 0.760 | 0.939 | 31.9% |
| urology | 0.912 | 0.806 | 1.032 | 33.6% |
