# Supplementary material for: GFR estimation is complicated by a high incidence of non-steady-state serum creatinine concentrations at the emergency department
Source: PLoS One. 2021 Dec 29;16(12):e0261977. doi: 10.1371/journal.pone.0261977 (PMC8716053; doi:10.1371/journal.pone.0261977)
Supplement: S7 Table — *: p-value < 0.001. (DOCX) [file pone.0261977.s007.docx]

S6 Table. Odds ratio for each emergency department (ED) specialism compared with the nephrology ED specialism in respect to a non-steady-state serum creatinine (SCr) between SCr-ED and SCr-H1. *: p-value < 0.001

| ED specialism | Odds ratio | 95% CI  lower bound | 95% CI  upper bound | Percentage  non-steady-state SCr |
| --- | --- | --- | --- | --- |
| Intercept (nephrology)* | 1.000 |  |  | 21.6% |
| cardiology | 1.136 | 0.959 | 1.348 | 23.9% |
| gastroenterology | 1.361 | 1.108 | 1.671 | 27.3% |
| internal medicine* | 1.690 | 1.445 | 1.983 | 31.8% |
| lung* | 1.592 | 1.319 | 1.925 | 30.5% |
| neurology* | 1.588 | 1.339 | 1.889 | 30.5% |
| other | 1.364 | 0.954 | 1.928 | 27.4% |
| surgical* | 1.540 | 1.306 | 1.821 | 29.8% |
| urology | 1.162 | 0.938 | 1.440 | 24.3% |
