# Supplementary figures and images for: GFR estimation is complicated by a high incidence of non-steady-state serum creatinine concentrations at the emergency department
Source: PLoS One. 2021 Dec 29;16(12):e0261977. doi: 10.1371/journal.pone.0261977 (PMC8716053; doi:10.1371/journal.pone.0261977)

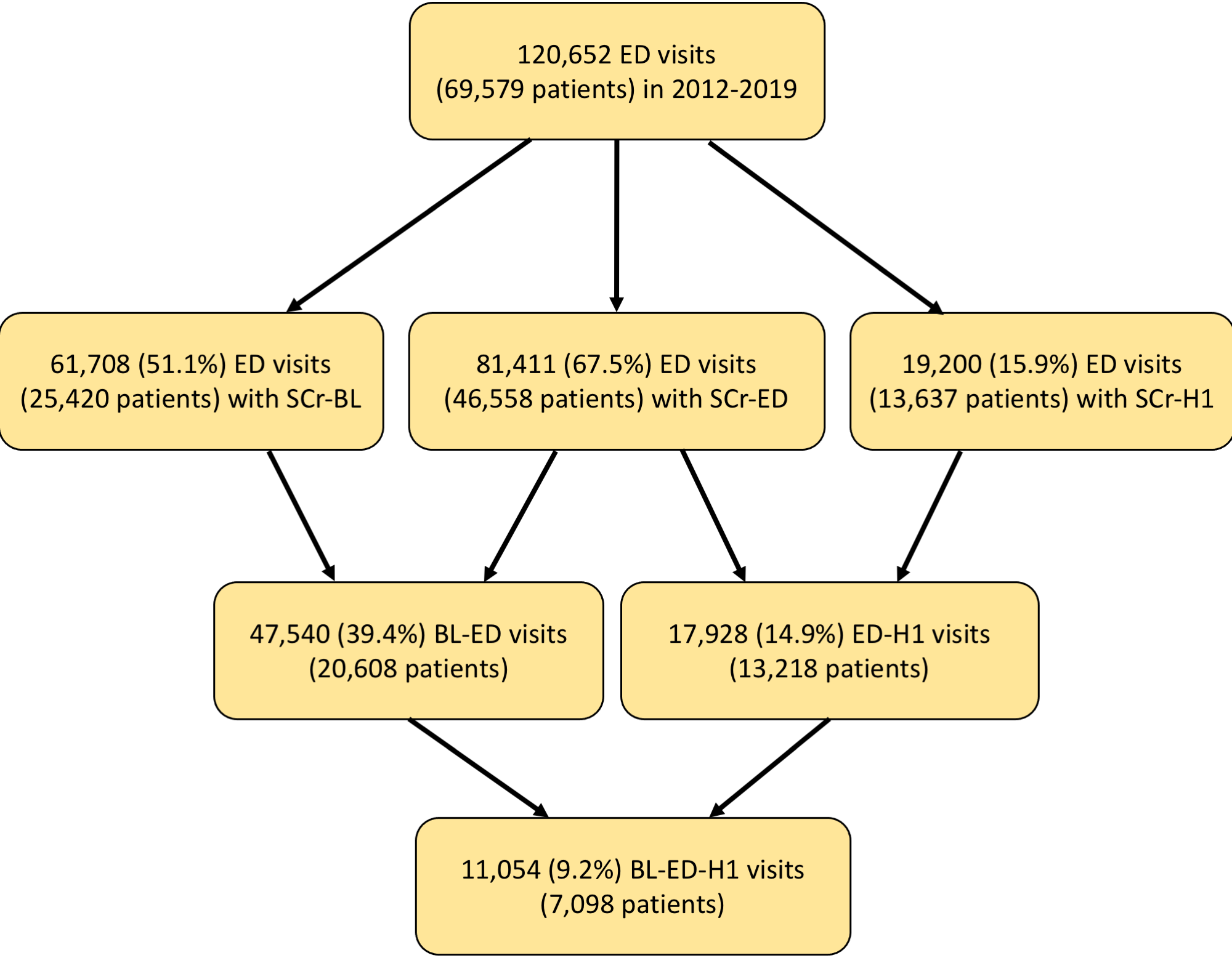

Supplement: S1 Fig — Percentages computed from the total number of ED visits. (TIFF) [file pone.0261977.s008.tiff]

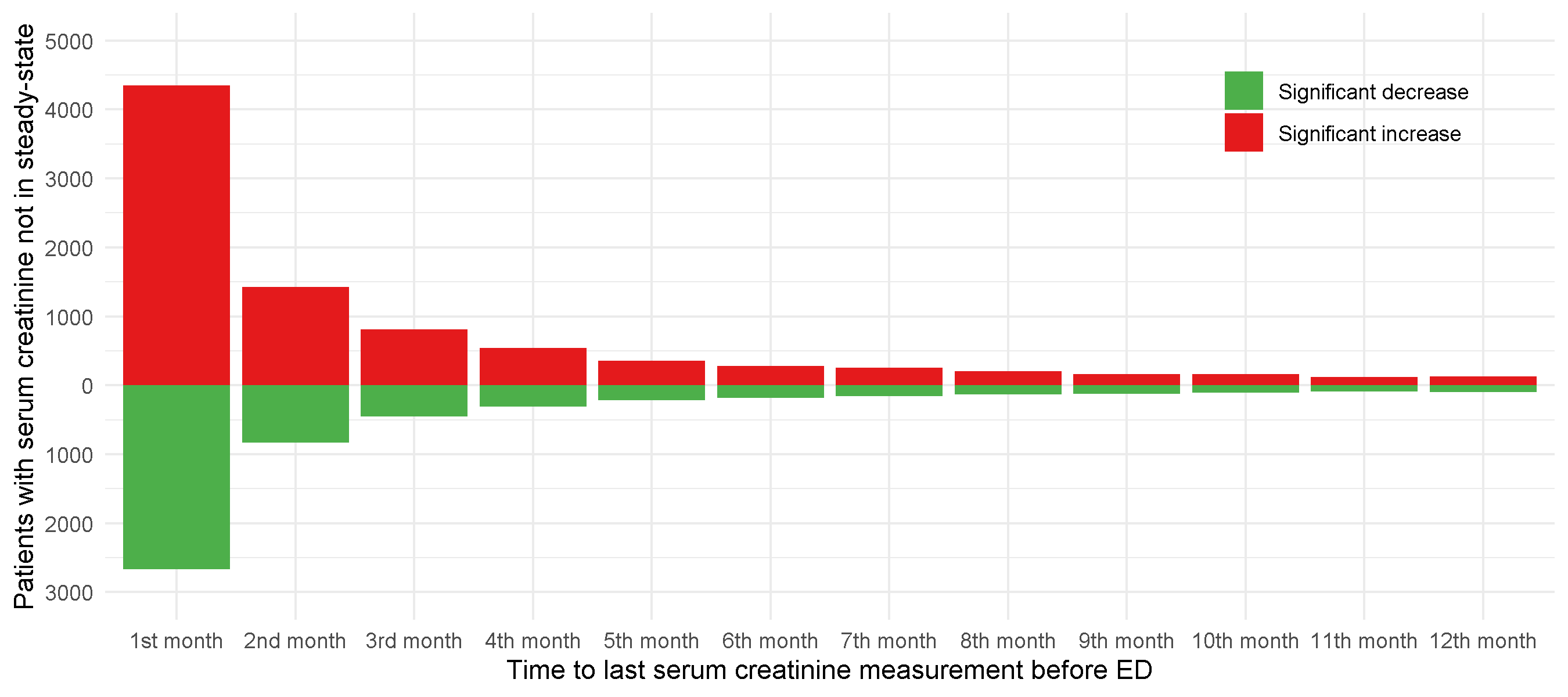

Supplement: S2 Fig — Patients are grouped based on the time to last creatinine measurement before emergency department (ED) visit. (TIFF) [file pone.0261977.s009.tiff]
